# COVID-19 Changed the Injury Patterns of Hospitalized Patients

**DOI:** 10.1017/S1049023X21000285

**Published:** 2021-03-01

**Authors:** Michael Rozenfeld, Kobi Peleg, Adi Givon, Miklosh Bala, Gad Shaked, Hany Bahouth, Moran Bodas

**Affiliations:** 1. Israel National Centre for Trauma and Emergency Research, Gertner Institute, Tel Hashomer, Israel; 2. Tel-Aviv University, Faculty of Medicine, School of Public Health, Tel-Aviv, Israel; 3. Trauma Unit, Hadassah Medical Center, Jerusalem, Israel; 4. Trauma Unit, Soroka Medical Center, Beersheba, Israel; 5. Trauma Unit, Rambam Medical Center, Haifa, Israel

**Keywords:** COVID-19, injury patterns, injury severity, lockdown, surgery, COVID-19, coronavirus disease 2019, ED, emergency department, ICU, intensive care unit, INTR, Israeli National Trauma Registry, ISS, Injury Severity Score, LOS, length of stay, RTC, road traffic collision

## Abstract

**Introduction:**

Injury patterns are closely related to changes in behavior. Pandemics and measures undertaken against them may cause changes in behavior; therefore, changes in injury patterns during the coronavirus disease 2019 (COVID-19) outbreak can be expected when compared to the parallel period in previous years.

**Study Objective:**

The aim of this study was to compare injury-related hospitalization patterns during the overall national lockdown period with parallel periods of previous years.

**Methods:**

A retrospective study was completed of all patients hospitalized from March 15 through April 30, for years 2016-2020. Data were obtained from 21 hospitals included in the national trauma registry during the study years. Clinical, demographic, and circumstantial parameters were compared amongst the years of the study.

**Results:**

The overall volume of injured patients significantly decreased during the lockdown period of the COVID-19 outbreak, with the greatest decrease registered for road traffic collisions (RTCs). Patients’ sex and ethnic compositions did not change, but a smaller proportion of children were hospitalized during the outbreak. Many more injuries were sustained at home during the outbreak, with proportions of injuries in all other localities significantly decreased. Injuries sustained during the COVID-19 outbreak were more severe, specifically due to an increase in severe injuries in RTCs and falls. The proportion of intensive care unit (ICU) hospitalizations did not change, however more surgeries were performed; patients stayed less days in hospital.

**Conclusions:**

The lockdown period of the COVID-19 outbreak led to a significant decrease in number of patients hospitalized due to trauma as compared to parallel periods of previous years. Nevertheless, trauma remains a major health care concern even during periods of high-impact disease outbreaks, in particular due to increased proportion of severe injuries and surgeries.

## Introduction

Injury patterns are known to be closely related to human behavior, both injury preventive and inducing.^1-3^ A major change in behavior patterns of the public, such as lockdown during epidemics, is likely to lead to changes in the regularly observed injury patterns. In turn, these changes can influence the ability of the health care system to deal with the injury burden.^4-7^

The new coronavirus disease 2019 (COVID-19) crisis had a direct impact on public behavior patterns both through restrictions enforced by the authorities (eg, lockdown and self-isolation orders) and self-inflicted due to an elevated threat perception by the public (fear to go outside and meet other people, change in shopping patterns, and so on).^8-10^ To name a few examples, the ban on social gatherings changed recreation patterns, enforced lockdown kept people at home, restriction on public transportation may have forced some people to resort to other means of commute, closure of education institutes kept students away from educational frameworks, and lay-offs and unpaid leaves removed people from workplaces.^8,11,12^ Such a significant change in public behavior may shift the injury risk patterns of individuals, and therefore may have an impact on actual injury.

In Israel, the first restrictions imposed onto the public were issued in the middle of March 2020, with the following timeline: after stating on March 10^th^ that all individuals returning from abroad were required to self-isolate at home for 14 days, the Ministry of Health published on March 15^th^ a list of restrictions to be enacted in the days following.^13^ On March 19^th^, first restrictions to prohibit outdoor activities, with the exception of “essential work,” were put into action. On March 25^th^, public gatherings and public transportation were terminated. On April 26^th^, some restrictions were removed, including opening of street stores and barbershops. On May 4^th^-5^th^, the majority of normal activities were reinstated.^14^ The overall national lockdown period could be thus recognized as starting around the middle of March and ending at the end of April.

This study’s objective was to explore differences in injury and hospitalization patterns due to traumatic injury in Israel during the overall national lockdown period, in comparison to the parallel periods of previous years.

## Methods

This is a retrospective study of all patients included in the Israeli National Trauma Registry (INTR) during the period of 2016-2020. The INTR contains information concerning trauma patients hospitalized in 21 hospitals, including all six Level I trauma centers in Israel. The INTR records data from all trauma patients with an ICD-9-CM diagnosis code between 800.0 and 959.9 who were hospitalized due to injury, following emergency department (ED) visit, including those who died in the ED or were transferred to another hospital. The INTR documents more than 90% of all trauma casualties and 98% of the severely injured trauma casualties. The INTR does not include patients who were declared dead at the scene or on arrival at the hospital or discharged from ED. The data are collected at the hospitals by dedicated trauma registrars, monitored by the trauma coordinator, and are under the responsibility of the trauma unit director. The data are entered into a computerized system and transmitted with no identifying details to the central database managed by the Israel National Center for Trauma and Emergency Medicine Research at the Gertner Institute (Tel Hashomer, Israel). After the data are received from the hospital and entered into the central database, logic and other tests are performed to ensure quality and completeness. Missing, unclear, or erroneous data are corrected and completed. When missing information is detected, a query is sent to the hospital to fill it in. For each patient in the INTR, there are approximately 150 variables providing demographic data, detailed injury data (circumstances/mechanism), information on prehospital treatment, treatment in the department of emergency medicine, operations, diagnosis (according to the ICD-9-CM and AIS), and destination upon discharge.

This study was approved by the Institutional Ethical Committee of The Sheba Medical Center (Ramat Gan, Israel), No.: 5138-18-SMC.

### Study Population

Out of each study year, a subset of patients injured from March 15 through April 30 was singled out in order to correspond to the lockdown period during the pandemic of 2020. Patients transferred between hospitals were included only in the receiving hospital in order to prevent double counting of them in the study population. Altogether, 25,712 patients met the inclusion criteria. The analyzed data included patient demographic details, the circumstantial parameters of the injury event, injury severity, hospital resources utilization, and clinical outcomes.

### Study Variables

The main independent variable was the year of hospitalization. All other parameters were compared by this factor.

Injury severity was represented by Injury Severity Score (ISS) and was analyzed both by four groups (minor: 1-8, moderate: 9-14, severe: 16-24, and critical: 25-75) and two groups (1-14, 16-75). Note that ISS = 15 is mathematically impossible to achieve in the way ISS is computed. Injury-specific parameters included the identity of injured body regions and the type of sustained trauma. Parameters for utilization of hospital resources were intensive care unit (ICU) admission and performance of any surgery, in general and within the first 24 hours following hospitalization. Length of stay (LOS) longer than two weeks and in-hospital mortality were the measures for clinical outcomes.

Circumstantial parameters included injury mechanism, location of the event, and the type of road user for road traffic collision (RTC) victims. Patients’ sex, ethnicity, and age were also considered in the analysis. Ethnicity was defined by membership of either the Jewish majority or any Non-Jewish (mostly Arab-speaking) minority. Age was divided into five groups: children aged 0-14, young adults aged 15-29, adults aged 30-44 and 45-59, and senior citizens aged 60 and older.

### Statistical Analyses

In every comparison, a significant change in year 2020 as compared to other years was sought. Chi-square test was utilized for all comparisons. A value of P <.05 was considered to be statistically significant. SAS statistical software version 9.4 was used for data analysis (SAS Institute; Cary, North Carolina USA). Patients or the public were not involved in the design, conduct, reporting, or dissemination plans of this research.

## Results

**Figure 1.**
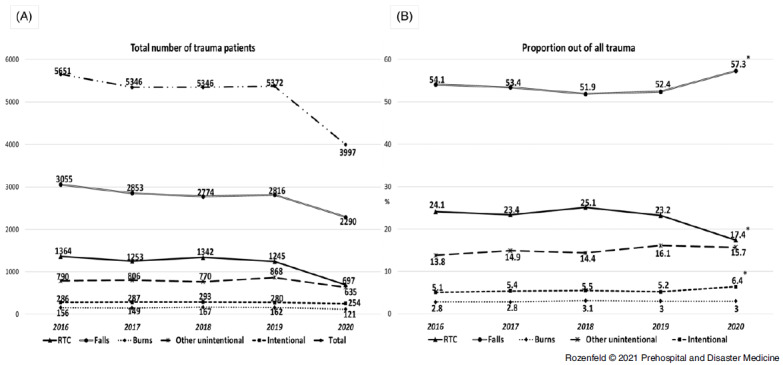
**(A)** Trends of Hospitalizations due to Trauma Injuries According to Injury Mechanism; **(B)** Trends of Proportion of Each Injury Mechanism Out of All Trauma Patients (March 15-April 30 of every studied year). Note: Presented differences of year 2020 compared to previous years are significant (Chi-Square sig.<.05). Abbreviation: RTC, road traffic collision.

An observable decrease was seen in the number of hospitalizations due to most injury mechanisms, as well as an overall decrease in the number of trauma hospitalizations of approximately 26% (Figure 1, A). The only mechanism where the decrease in numbers was not observed was intentional injuries. Additionally, a significant change in the relative proportions of different injury mechanisms was observed, including a decrease in RTC-related injuries and a growth of injuries from falls and intentional injuries (Figure 1, B).

Table 1 presents the demographic and the circumstantial characteristics of the study population compared throughout studied years. The overall number of injured patients remained relatively stable in March-April of 2016-2019 and decreased drastically in 2020 during the COVID-19 outbreak.

**Table 1.** Distribution (n (%) 95% CI) of Demographic and Circumstantial Characteristics of Injury in Study Population (N = 25,712) for the Period of March 15-April 30 of Every Studied Year

	2016	2017	2018	2019	2020
**Sex**					
Males	3398 (60.1) (58.8–61.4)	3185 (59.6) (58.2–60.9)	3263 (61.0) (59.7–62.3)	3160 (58.8) (58.5–60.1)	2447 (61.2) (59.7–62.7)
Females	2253 (39.9) (38.6–41.2)	2161 (40.4) (39.1–41.7)	2083 (39.0) (37.6–40.3)	2212 (41.2) (39.9–42.5)	1550 (38.8) (37.3–40.3)
**Ethnicity**					
Jewish	3838 (68.8) (66.7–69.1)	3591 (68.1) (65.9–68.4)	3648 (68.2) (67.0–69.5)	3725 (69.3) (68.1–70.6)	2678 (67.0) (65.5–68.5)
Non-Jewish	1761 (31.2) (30.0–32.4)	1705 (31.9) (30.6–33.2)	1698 (31.8) (30.5–33.0)	1647 (30.7) (29.4–31.9)	1319 (33.0) (31.5–34.5)
**Age**					
0–14	1470 (26.0) (24.9–27.2)	1329 (24.9) (23.7–26.0)	1283 (24.0) (22.9–25.2)	1264 (23.5) (22.4–24.7)	814 ***(20.4)*** ***(19.1–21.6)***
15–29	1103 (19.5) (18.5–20.6)	1062 (19.9) (18.8–21.0)	1111 (20.8) (19.7–21.9)	1024 (19.1) (18.0–20.2)	773 (19.3) (18.1–20.6)
30–44	694 (12.3) (11.4–13.2)	630 (11.8) (10.9–12.7)	696 (13.0) (12.1–13.9)	687 (12.8) (11.9–13.7)	502 (12.6) (11.5–13.6)
45–59	550 (9.7) (9.0–10.5)	532 (10.0) (9.2–10.8)	549 (10.3) (9.5–11.1)	583 (10.9) (10.0–11.7)	471 (11.8) (10.8–12.8)
60+	1834 (32.5) (31.2–33.7)	1793 (33.5) (32.3–34.8)	1707 (31.9) (30.7–33.2)	1814 (33.8) (32.5–35.0)	1437 ***(35.9)*** (34.5–37.5)
**Location**					
Residence	2425 (42.9) (41.6–44.2)	2239 (41.9) (40.5–43.2)	2131 (39.9) (38.5–41.2)	2161 (40.2) (38.9–41.5)	2280 ***(57.0)*** ***(55.5–58.6)***
Sport Center	148 (2.6) (2.2–3.1)	149 (2.8) (2.4–3.3)	120 (2.2) (38.6–41.2)	135 (2.5) (2.1–3.0)	20 ***(0.5)*** ***(0.3–0.7)***
School	107 (1.9) (1.5–2.3)	99 (1.9) (1.4–2.2)	120 (2.2) (1.9–2.7)	97 (1.8) (1.5–2.2)	1 ***(0.03)*** ***(0.01–0.1)***
Public Recreation	152 (2.7) (2.3–3.1)	142 (2.7) (2.2–3.1)	143 (2.7) (2.3–3.1)	154 (2.9) (2.4–3.3)	51 ***(1.3)*** ***(0.9–1.7)***
Playground	134 (2.4) (2.0–2.8)	116 (2.2) (1.7–2.6)	163 (3.0) (2.6–3.5)	118 (2.2) (1.8–2.6)	32 ***(0.8)*** ***(0.5–1.1)***
Other	2685 (47.5) (46.2–48.8)	2601 (48.5) (47.3–50.0)	2669 (50) (48.6–51.3)	2707 (50.4) (49.0–51.7)	1613 ***(40.4)*** ***(38.8–41.9)***
**Country Region**					
Center	2920 (51.7) (50.4–53.0)	2747 (51.4) (50.0–52.7)	2708 (50.7) (49.3–52.0)	2812 (52.4) (51.0–53.7)	1899 ***(47.5)*** ***(45.9–49.1)***
South	646 (11.4) (10.6–12.3)	711 (13.3) (12.4–14.2)	727 (13.6) (12.7–14.5)	703 (13.1) (12.2–14.0)	722 ***(18.1)*** ***(16.9–19.3)***
North	2085 (36.9) (35.6–38.2)	1888 (35.3) (34.0–36.6)	1911 (35.7) (34.5–37.0)	1856 (34.5) (33.3–35.8)	1376 (34.4) (32.9–35.9)
**Day of Week**					
Weekday	4089 (72.4) (71.2–73.5)	3805 (71.2) (69.9–72.4)	3849 (72.0) (70.8–73.2)	3898 (72.5) (71.3–73.7)	3059 ***(76.5)*** ***(75.2–77.8)***
Weekend^a^	1562 (27.6) (26.5–28.8)	1541 (28.8) (27.6–30.1)	1497 (28.0) (26.8–29.2)	1474 (27.5) (26.2–28.6)	938 ***(23.5)*** ***(22.2–24.8)***
**Time of Day**					
23:00–6:59	770 (13.6) (12.7–14.5)	753 (14.1) (13.2–15.0)	766 (14.3) (13.4–15.3)	774 (14.4) (13.5–15.4)	577 (14.4) (13.4–15.6)
07:00–14:59	2061 (36.5) (35.2–37.7)	1994 (37.3) (36.0–38.6)	1988 (37.2) (35.9–38.5)	2048 (38.1) (36.8–39.4)	1535 (38.4) (36.9–39.9)
15:00–22:59	2820 (49.9) (48.6–51.2)	2599 (48.6) (47.3–50.0)	2592 (48.5) (47.1–49.8)	2549 (47.5) (46.1–48.8)	1885 (47.2) (45.6–48.7)
**Total N**	5651	5346	5346	5372	***3997***

Note: Significant differences of year 2020 compared to previous years (Chi-Square sig.<.05) are marked by bold italic font.

a Weekends in Israel span over Friday and Saturday. Week starts on Sunday.

In terms of age composition, there was a significant decrease in the number of injured children aged 0-14 years old in 2020 corresponding to an increase of injuries among senior citizens older than 60. No change was detected in other age groups. The relative proportion of males and females, as well as that of the Jewish majority and Non-Jewish ethnical minority, remained unchanged throughout studied years.

The greatest change was observed concerning location of the injury event: a significant increase was observed in injuries at home, while the proportion of injuries in all other locations decreased. Another location-related change in 2020 was observed on a macro level: a significantly larger proportion of trauma patients were hospitalized in the Southern region of the country, at the expense of less patients in the larger Central region. A smaller proportion of patients were hospitalized on weekends during the lockdown period of 2020, however no change was detected in the hospitalization time of day.

The injuries in March-April 2020, though of smaller volume compared to previous years, were more severe (Table 2). The greatest increase took place in the proportion of moderate (ISS 9-14) and severe (ISS 16-24) injuries. While minor injuries (ISS 1-8) in 2020 accounted for 57%, this proportion ranged between 61% to 63% in previous years. The frequency of penetrating trauma increased on the expense of blunt trauma. There was little change in proportion of injuries to most body regions, however, a significant decline in abdomen injuries was observed, as well as a significant increase in injuries to extremities, especially hip fractures.

**Table 2.** Distribution (n (%) 95% CI) of Injury Parameters, Severity, Hospitalization Resources Utilization, and Clinical Outcomes of Study Population for the Period of March 15-April 30 of Every Studied Year

	2016	2017	2018	2019	2020
**Trauma Type**					
Blunt Only	4854 (85.9) (84.0–86.8)	4506 (84.2) (83.3–85.2)	4541 (84.9) (83.9–85.9)	4516 (84.1) (83.1–85.0)	3284 ***(82.2)*** ***(80.9–83.3)***
Penetrating Only	464 (8.2) (7.5–9.0)	524 (9.8) (9.0–10.6)	443 (8.3) (7.6–9.1)	518 (9.6) (8.9–10.5)	462 ***(11.6)*** ***(10.6–12.6)***
Burn Only	161 (2.9) (2.4–3.3)	155 (2.9) (2.5–3.4)	178 (3.3) (2.9–3.8)	185 (3.4) (3.0–4.0)	126 (3.1) (2.6–3.7)
Multiple/Other	172 (3.0) (2.6–3.5)	161 (3.1) (2.6–3.5)	184 (3.4) (3.0–4.0)	152 (2.8) (2.4–3.3)	125 (3.1) (2.6–3.7)
**Injured Body Region^a^**					
Head	1391 (24.6) (23.5–25.8)	1137 (21.3) (20.2–22.4)	1201 (22.5) (21.3–23.6)	1203 (22.4) (21.3–23.5)	868 (21.7) (20.4–23.0)
Face	722 (12.8) (11.9–13.7)	669 (12.5) (11.6–13.4)	711 (13.3) (12.4–14.2)	704 (13.1) (12.2–14.0)	485 (12.1) (11.1–13.2)
Neck	59 (1.0) (0.8–1.3)	75 (1.4) (1.1–1.8)	86 (1.6) (1.3–2.0)	62 (1.2) (0.9–1.5)	35 (0.9) (0.6–1.2)
Chest	623 (11.0) (10.2–11.9)	596 (11.2) (10.3–12.0)	610 (11.4) (10.6–12.3)	601 (11.2) (10.4–12.1)	440 (11.0) (10.0–12.0)
Abdomen	629 (11.1) (10.3–12.0)	643 (12.0) (11.2–12.9)	674 (12.6) (11.7–13.5)	642 (12.0) (11.1–12.8)	362 ***(9.1)*** ***(8.2–10.0)***
Spine	303 (5.4) (4.8–6.0)	259 (4.8) (4.3–5.4)	277 (5.2) (4.6–5.8)	331 (6.2) (5.5–6.8)	216 (5.4) (4.7–6.1)
Upper Extremities	1361 (24.1) (23.0–25.2)	1357 (25.4) (24.2–26.6)	1404 (26.3) (25.1–27.5)	1371 (25.5) (24.4–26.7)	1109 ***(27.8)*** ***(26.4–29.2)***
Lower Extremities	2266 (40.1) (38.8–41.4)	2142 (40.1) (38.7–41.4)	2194 (41.0) (39.7–42.4)	2174 (40.5) (39.1–41.8)	1785 ***(44.7)*** ***(43.1–46.2)***
Hip Fracture	740 (13.1) (12.2–14.0)	749 (14.0) (13.1–15.0)	760 (14.2) (13.3–15.2)	735 (13.7) (12.8–14.6)	674 ***(16.9)*** ***(15.7–18.1)***
**Injury Severity**					
ISS 1–8	3564 (63.1) (61.8–64.3)	3361 (62.8) (61.6–64.2)	3278 (61.3) (60.0–62.6)	3400 (63.3) (62.0–64.6)	2272 ***(56.8)*** ***(55.3–58.4)***
ISS 9–14	1594 (28.2) (27.0–29.4)	1547 (29.0) (27.7–30.2)	1561 (29.2) (28.0–30.4)	1489 (27.7) (26.5–28.9)	1309 ***(32.8)*** ***(31.3–34.2)***
ISS 16–24	292 (5.2) (4.6–5.8)	262 (4.9) (4.3–5.5)	304 (5.7) (5.1–6.3)	299 (5.6) (5.0–6.2)	268 ***(6.7)*** ***(5.9–7.5)***
ISS 25–75	200 (3.5) (3.1–4.0)	176 (3.3) (2.8–3.8)	203 (3.8) (3.3–4.3)	184 (3.4) (2.9–3.9)	148 (3.7) (3.1–4.3)
**Hospital Resources**					
ICU Utilization	342 (6.1) (5.4–6.7)	301 (5.6) (5.0–6.3)	324 (6.1) (5.4–6.7)	298 (5.6) (4.9–6.2)	253 (6.3) (5.6–7.2)
Surgery	2143 (37.9) (36.6–39.2)	2063 (38.6) (37.3–39.9)	2163 (40.5) (39.1–41.8)	2129 (39.6) (38.3–40.9)	1972 ***(49.3)*** ***(47.8–50.9)***
Surgery within 24 Hours	1199 (21.2) (20.2–22.3)	1155 (21.6) (20.5–22.7)	1256 (23.5) (22.4–24.6)	1171 (21.8) (20.7–22.9)	1266 ***(31.7)*** ***(30.2–33.1)***
**Outcomes**					
LOS 15+	389 (6.9) (6.2–7.6)	372 (7.0) (6.3–7.7)	333 (6.2) (5.6–6.9)	322 (6.0) (5.4–6.7)	173 ***(4.3)*** ***(3.7–5.0)***
Mortality	74 (1.3) (1.0–1.6)	72 (1.3) (1.1–1.7)	77 (1.4) (1.1–1.8)	64 (1.2) (0.9–1.5)	62 (1.6) (1.2–2.0)
**Total N**	5651	5346	5346	5372	***3997***

Note: Significant differences of year 2020 compared to previous years (Chi-Square sig.<.05) are marked by bold italic font.

Abbreviations: ISS, Injury Severity Score; ICU, intensive care unit; LOS, length of stay.

a Same patient may have injuries to more than one body region.

Table 2 shows the utilization of hospital resources and the clinical outcomes due to trauma injuries in each studied year. While the levels of admission to the ICU remained stable, a significant increase in performed surgeries was detected. A significantly smaller proportion of patients’ LOS in hospitals was longer than two weeks in 2020, while in-hospital mortality did not change.

**Figure 2.**
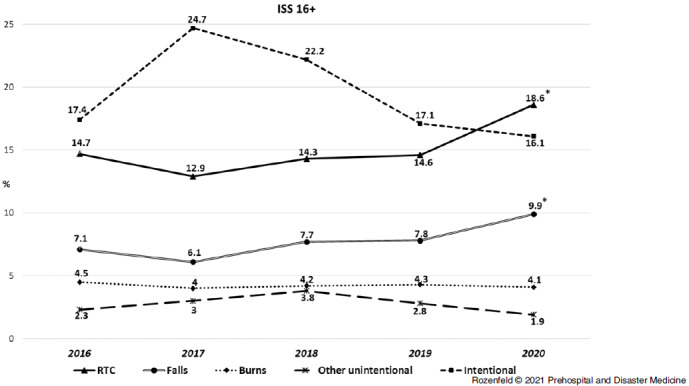
Injury Severity Trends According to Injury Mechanisms (Proportion of ISS 16+ in the Period of March 15-April 30 of Every Studied Year). Note: Presented differences of year 2020 compared to previous years are significant (Chi-Square sig.<.05). Abbreviations: ISS, Injury Severity Score; RTC, road traffic collision.

In order to uncover the factors that led to increase in injury severity in March-April 2020, the trend of severe and critical injuries (ISS 16-75) was analyzed for every injury mechanism (Figure 2). A significant increase in injury severity in 2020 was detected for patients injured in RTCs and falls. The trends remained stable for victims of burns and other unintentional injury, while victims of intentional injury (violence and suicide) experienced a peak of injury severity in 2017-2018 and returned to previous levels in 2019-2020.

As for RTC patients, both a decrease in number of hospitalizations and an increase in injury severity was detected. Therefore, an in-depth analysis of this sub-population was pursued (Table 3). Taking into consideration that the overall number of RTC patients cut by one-half in 2020, significant changes were observed in all demographic and circumstantial parameters. In terms of road users who were injured, there was a significant increase in proportion of injuries of drivers of motorcycles, bicycles, animal carriages, and electric bicycles/scooters. The proportion of vehicle passengers and pedestrian has decreased; however, the proportion of four-wheeled vehicle drivers did not change.

**Table 3.** Distribution (n (%) 95% CI) of the RTC Population Characteristics for the Period of March 15-April 30 of Every Studied Year

	2016	2017	2018	2019	2020
**Road User**					
Vehicle Driver	300 (22.0) (19.8–24.3)	285 (22.7) (20.4–25.2)	307 (22.9) (20.6–25.2)	307 (24.7) (22.3–27.1)	154 (22.1) (19.1–25.4)
Vehicle Passenger	251 (18.4) (16.4–20.6)	211 (16.8) (14.8–19.0)	225 (16.8) (14.8–18.9)	224 (18.0) (15.9–20.2)	67 ***(9.6)*** ***(7.5–12.0)***
Motorcycle	187 (13.7) (11.9–15.6)	169 (13.5) (11.6–15.5)	206 (15.3) (13.5–18.4)	164 (13.1) (11.3–15.2)	127 ***(18.2)*** ***(15.4–21.3)***
Animal Carriage	27 (2.0) (1.3–2.9)	35 (2.8) (1.9–3.9)	33 (2.5) (1.7–3.4)	22 (1.8) (1.1–2.7)	32 ***(4.6)*** ***(3.2–6.4)***
Bicycle	227 (16.6) (14.7–18.7)	209 (16.7) (14.7–18.9)	205 (15.3) (13.4–17.3)	162 (13.0) (11.2–15.0)	130 ***(18.6)*** ***(15.8–21.7)***
Electric Two-Wheeled	69 (5.1) (4.0–6.4)	80 (6.4) (5.1–7.9)	104 (7.8) (6.4–9.3)	98 (7.9) (6.4–9.5)	68 ***(9.8)*** ***(7.7–12.2)***
Pedestrian	284 (20.8) (18.7–23.1)	234 (18.7) (16.5–20.9)	230 (17.1) (15.2–19.3)	238 (19.1) (17.0–21.4)	102 ***(14.6)*** ***(12.1–17.5)***
Other	19 (1.4) (0.8–2.2)	30 (2.4) (1.6–3.4)	32 (2.4) (1.6–3.3)	30 (2.4) (1.6–3.4)	17 (2.5) (1.5–3.9)
**Sex**					
Males	910 (66.7) (64.1–69.2)	845 (67.4) (64.8–70.0)	929 (69.2) (66.7–71.7)	803 (64.5) (61.8–67.2)	546 ***(78.3)*** ***(75.1–81.3)***
Females	454 (33.3) (30.8–35.9)	408 (32.6) (30.0–35.2)	413 (30.8) (28.3–33.3)	442 (35.5) (32.8–38.2)	151 ***(21.7)*** ***(18.7–24.9)***
**Ethnicity**					
Jewish	906 (66.4) (63.8–68.9)	798 (63.7) (60.9–66.3)	870 (64.8) (62.2–67.4)	833 (66.9) (64.2–69.5)	380 ***(54.5)*** ***(50.7–58.3)***
Non-Jewish	458 (33.6) (31.2–36.1)	455 (36.3) (33.6–39.0)	472 (35.2) (32.6–38.8)	412 (33.1) (30.4–35.8)	317 ***(45.5)*** ***(41.7–49.3)***
**Age**					
0–14	325 (23.8) (21.6–26.2)	284 (22.7) (20.4–25.1)	267 (19.9) (17.8–22.1)	260 (20.9) (18.7–23.2)	124 ***(17.8)*** ***(15.0–20.8)***
15–29	458 (33.6) (31.1–36.1)	437 (34.9) (32.2–37.6)	472 (35.2) (32.6–37.8)	436 (35.0) (32.4–37.7)	292 ***(41.9)*** ***(38.2–45.7)***
30–44	212 (15.5) (13.7–17.6)	220 (17.6) (15.5–19.8)	243 (18.1) (16.7–20.3)	209 (16.8) (14.7–19.0)	118 (16.9) (14.2–19.9)
45–59	178 (13.0) (11.3–14.9)	133 (10.6) (9.0–12.4)	179 (13.3) (11.6–15.3)	148 (11.9) (10.1–13.8)	74 (10.6) (8.4–13.1)
60+	191 (14.0) (12.2–16.0)	179 (14.3) (12.4–16.3)	181 (13.5) (11.7–15.4)	192 (15.4) (13.5–17.5)	89 (12.8) (10.4–15.5)
**Total N**	1364	1253	1342	1245	***697***

Note: Significant differences of year 2020 compared to previous years (Chi-Square sig.<.05) are marked by bold italic font.

Abbreviation: RTC, road traffic collision.

In terms of demographic composition, a significant increase in the proportion of males and Non-Jewish ethnical minorities among RTC casualties took place. Concerning age, there were more injured young adult road users aged 15-29, while the proportion of injured children had decreased.

## Discussion

This study found that the restrictive measures imposed by authorities in order to control the COVID-19 pandemics introduced significant changes into the patterns of traumatic injury on a nation-wide basis. The overall volume of hospitalizations due to injury, especially due to RTCs, steeply declined during the lockdown period in comparison to parallel periods of previous years. The characteristics of patients who were hospitalized with injury during the 2020 COVID-19 outbreak were different in many aspects in relation to the previous years.

The most important feature of imposing lockdown of the population is the restriction of movement.^8,9^ With people not leaving home for work, studying, or recreation due to lockdown, the whole spectrum of potential risks for traumatic injuries that existed in those activities (sports injuries, street violence, work- and school-related injuries, and RTCs) is suddenly minimized.^8,11,15^ The place of residence becomes the place of highest exposure due the amount of time spent there.^8,11^ This is exactly what was found in this study: a significant increase in proportion of injuries at home residence, while the proportion of injuries in all other locations drastically decreased. However, the degradation of the overall risk for traumatic injury and the displacement of most risks to the home environment did not change the many patterns that are usually observed in trauma populations. The demographic composition of hospitalized patients remained largely the same in the middle age groups, with children aged 0-14 being more protected due to their decreased exposure to outside risks and a proportion increase in the injury of senior citizens aged 60 and older. There was a significant decline in the volume of RTC injuries and an increase in injuries from falls and intentional injuries; however, the relative proportion of other injury mechanisms remained unchanged.

A partial explanation for the reduced impact of lockdown on the demographic and circumstantial characteristics of hospitalized patients, despite the decrease in their volume and exposure, are two integral traits of trauma population – a two-tailed age distribution and falls being a dominant injury mechanism.^16,17^ Trauma patients are known to be either older or very young, with persons of middle age less prone to injury.^16^ For senior citizens (35% of the studied population), falls are the greatest risk. A significant part of falls take place at home, thus even decreased exposure to such risky environments in terms of falls as steps and public transportation will not make them safer.^17^ This understanding is corroborated by the significant increase in hip fractures found in this study. For the very young children, the greatest risk is not just from falls but from falling objects, which remained unchanged; however, there are many risks of falling at home for them as well.^16,17^

A similar process of outdoor risks being replicated at home could also be hypothesized in regard to burns and intentional injuries. A decrease in exposure to industrial burns could be negated by an increased risk of burns indoors due to the increase in cooking at home, and with small children present, this becomes a significant risk.^18,19^ Lower exposure to street and political violence is balanced by higher risk of partner or parental violence, as well as self-harm, with families being together most of the time, subjected to the widely discussed psychological impact of quarantine.^11^ In the current study, a significant proportional increase in intentional injuries was observed, corroborating this notion.

The only mechanism that could not be easily replicated to home environment is RTC, and this is where the greatest differences are found. Not only that the numerical decrease here was the most significant (more than 50%), but the demographic and the circumstantial characteristics of the patients have completely changed. In addition to decreased exposure of children, a significant increase was observed in hospitalization of younger males aged 15-29, especially members of Non-Jewish minority. Coupled with the found increased proportional incidence of drivers of two-wheeled transportation means, these finding may resonate the increased demand for delivery services during lockdown. As most Arab-speaking minorities in Israel live in rural environments,^20^ a significant increase in hospitalizations of animal transport drivers may suggest an attempt to cope with driving restrictions by turning to traditional non-motorized transport.

Interestingly, the influence of movement restrictions was clearly observed in the decreased proportion of hospitalized pedestrians and vehicle passengers, as the restrictions limited the number of allowed passengers to one, while the allowed walking distance was decreased to 100 meter from the place of residence.^13,14^ On the other hand, the relative proportion of hospitalized drivers of four-wheeled vehicles did not change, meaning that the roads where never clear of vehicles, as the essential personnel kept driving to work, while the supply of deliveries to the grocery store continued as usual.

The most interesting change in clinical aspects of the injury during pandemics was the increase in their severity. While the increase in the lethality of RTCs due to higher driving speeds on empty roads was widely reported,^15,21^ it is hard to provide a non-speculative explanation in regard to falls. However, this is an important aspect for the estimation of the change in the injury burden during pandemics: can the decrease in the overall volume of injuries alleviate the fact that hospitals have to deal with more complicated injury scenarios? The analysis of hospital resources utilization and clinical outcomes sheds some light on this issue.

Intensive care unit utilization and in-hospital mortality remained unchanged, most likely due to the fact that increase in severity did not involve critical injuries (ISS 25-75) and was mostly concentrated in moderate (ISS 9-14) and severe injuries (ISS 16-24). On the other hand, there was a significant increase in the volume of performed surgeries, especially in the first 24 hours. This could be a function of two factors: an increase in moderate and severely injured patients demanding surgery and the fact that due to the decline in the overall patient burden, more surgery teams could be available for elective and other surgeries.^4^ Additionally, the data suggest that during the COVID-19 outbreak, patients’ LOS were shorter at the hospital. In addition to faster surgery, it may be that some hospitals employed a conscious policy of sending the patients home into lockdown as fast as possible, so as to reduce the integrative contagion risks of the hospital environment and to make more hospital resources available for dealing with contingencies of the crisis.

## Limitations

The greatest limitation of this study is related to the systemic impact of COVID-19-related factors on the environment. It is therefore difficult to establish whether the found differences are an outcome of an actual change in incidence of different injury-causing scenarios, rather than of a reduced readiness of some patients to present to hospital due to infection concerns or of deliberate hospital policy not to hospitalize less severe cases. Nevertheless, the study faithfully presents the characteristics of patients who were hospitalized during the study period.

It is also important to remember that the INTR database compiles information from different hospitals, with some level of variation existing in the reported data. This may be considered a limitation to making national-level conclusions, however, this is common to most registries.

## Conclusions

This study shows that behavior changes induced by disease outbreaks and the involved lockdown restrictions had a significant impact on patterns of traumatic injury. Despite a significant decline in the overall volume of trauma hospitalizations, there was an increase in the proportion of moderate and severe injuries and in the need for surgery. This means that even during decrease in public activities caused by pandemic-induced lockdown, trauma wards should be kept a priority.

## References

[1] Cinnamon J , Schuurman N , Hameed SM. Pedestrian injury and human behavior: observing road-rule violations at high-incident intersections. PLoS ONE. 2011;6(6):e21063. 21698258 10.1371/journal.pone.0021063 PMC3115980

[2] Feyer A-M , Williamson AM , Cairns DR. The involvement of human behavior in occupational accidents: errors in context. Safety Science. 1997;25 (1-3):55–65.

[3] Cherry C , Leong KM , Wallen R , Buttke D. Risk-enhancing behaviors associated with human injuries from bison encounters at Yellowstone National Park, 2000–2015. One Health. 2018;6:1–6. 30069498 10.1016/j.onehlt.2018.05.003 PMC6066602

[4] Allevi F , Dionisio A , Baciliero U , et al. Impact of COVID-19 epidemic on maxillofacial surgery in Italy. Br J Oral Maxillofac Surg. 2020;58(6):692–697. 32414539 10.1016/j.bjoms.2020.04.035 PMC7196423

[5] Jespersen E , Holst R , Franz C , Rexen CT , Wedderkopp N. Seasonal variation in musculoskeletal extremity injuries in school children aged 6-12 followed prospectively over 2.5 years: a cohort study. BMJ Open. 2014;4(1):e004165. 10.1136/bmjopen-2013-004165 PMC3902503 24401728

[6] Røislien J , Søvik S , Eken T. Seasonality in trauma admissions – are daylight and weather variables better predictors than general cyclic effects? PLoS ONE. 2018;13(2):e0192568. 29425210 10.1371/journal.pone.0192568 PMC5806884

[7] Ashby K , Pointer S , Eager D , Day L. Australian trampoline injury patterns and trends. Aust N Z J Public Health. 2015;39(5):491–494. 26123781 10.1111/1753-6405.12404

[8] Brooks SK , Webster RK , Smith LE , et al. The psychological impact of quarantine and how to reduce it: rapid review of the evidence. Lancet. 2020;395:912–920. 32112714 10.1016/S0140-6736(20)30460-8 PMC7158942

[9] Serafini G , Parmigiani B , Amerio A , Aguglia A , Sher L , Amore M. The psychological impact of COVID-19 on the mental health in the general population. QJM. 2020;113(8):531–537. 10.1093/qjmed/hcaa201 PMC7337855 32569360

[10] Dubey S , Biswas P , Ghosh R , et al. Psychosocial impact of COVID-19. Diabetes Metab Syndr. 2020;14(5):779–788. 32526627 10.1016/j.dsx.2020.05.035 PMC7255207

[11] Olding J , Zisman S , Olding C , Fan K. Penetrating trauma during a global pandemic: changing patterns in interpersonal violence, self-harm and domestic violence in the Covid-19 outbreak. Surgeon. 2021;19(1):e9–e13. 32826157 10.1016/j.surge.2020.07.004 PMC7392113

[12] Bali RK , Chaudhry K. Maxillofacial surgery and COVID-19, the pandemic!! J Maxillofac Oral Surg. 2020;19(2):159–161. 32292253 10.1007/s12663-020-01361-8 PMC7148429

[13] Times of Israel Staff. No more daycare, restaurants, gyms or prayer quorums: the new virus regulations. *Times of Israel.* https://www.timesofisrael.com/no-more-daycare-restaurants-gyms-or-prayer-quorums-the-new-virus-regulations/. Accessed August 2, 2020.

[14] Times of Israel Staff. No more 100-meter limit; malls, libraries to reopen: all the eased regulations. *Times of Israel.* https://www.timesofisrael.com/malls-libraries-gyms-and-zoos-the-businesses-that-can-reopen-under-new-rules/. Accessed August 2, 2020.

[15] Masri L. Car crashes deadlier as drivers speed during lockdowns. *Reuters*. https://www.reuters.com/article/us-health-coronavirus-traffic-casualties/car-crashes-deadlier-as-drivers-speed-during-lockdowns-idUSKBN23X1OR. Accessed August 2, 2020.

[16] WHO. Falls Fact Sheet. WHO web site. https://www.who.int/news-room/fact-sheets/detail/falls. Accessed August 2, 2020.

[17] James SL , Lucchesi LR , Bisignano C , et al. The global burden of falls: global, regional and national estimates of morbidity and mortality from the Global Burden of Disease Study 2017. Inj Prev. 2020;26(Suppl 1):i3–i11. 31941758 10.1136/injuryprev-2019-043286 PMC7571347

[18] WHO. Burns Fact Sheet. WHO web site. https://www.who.int/news-room/fact-sheets/detail/burns. Accessed August 2, 2020.

[19] Haik J , Liran A , Tessone A , Givon A , Orenstein A , Peleg K. Burns in Israel: demographic, etiologic and clinical trends, 1997-2003. IMAJ. 2007;9(9):659–662. 17939628

[20] Magid A , Leibovitch-Zur S , Baron-Epel O. Increased inequality in mortality from road crashes among Arabs and Jews in Israel. Traffic Injury Prevention. 2015;16(1):42–47. 24679219 10.1080/15389588.2014.908289

[21] ETSC. The Impact of Covid-19 Lockdowns on Road Deaths in April 2020 (PIN Briefing). Brussels, Belgium: European Transport Safety Council; July 9, 2020.

